# Green Synthesis of 8‐Hydroxyquinoline Barium as Visible‐Light‐Excited Luminescent Material Using Mechanochemical Activation Method

**DOI:** 10.1002/gch2.201900052

**Published:** 2019-09-06

**Authors:** Junchen Liu, Xueming Zhong, Yuna Xu, Yanrong Li

**Affiliations:** ^1^ School of Environmental & Chemical Engineering Nanchang Hangkong University Nanchang 330063 China

**Keywords:** 8‐hydroxyquinoline barium, excited theories, green synthesis, mechanochemical activation method, visible‐light‐excited

## Abstract

Using high‐energy UV‐light to excite 8‐hydroxyquinoline barium (BaQ_2_) is a short slab for this emerging area of organic luminescent materials. However, using visible light to excite BaQ_2_ has not been reported. To solve this problem, this study proposes the mechanochemical activation method to synthesize luminescent material of visible‐light‐excited BaQ_2_. This research applies infrared spectroscopy, X‐ray diffraction (XRD), X‐ray photoelectron spectroscopy, scanning electron microscopy, energy dispersive spectroscopy, and fluorescence spectrometry to analyze the structure and luminescence properties. XRD shows that BaQ_2_ has a high crystallinity, small crystalline size, and high purity. According to the Scherrer equation, the mean particle size is 56 nm. The results of fluorescence spectrometry show that the excitation spectrum of the product is red‐shifted, and the maximum excitation wavelength is 408 nm. According to these results, the product has a high fluorescence and can be excited under visible light. This research explains the high efficiency of the mechanochemical‐activation method by thermodynamic and dynamic principles. This research also exemplifies luminescence theory of BaQ_2_ and at the microlevel explains the theory of visible‐light‐excited theory and the principle of luminescent intensity enhancement from the point of crystallography.

## Graphical abstract

**A new method for the synthesis of 8‐hydroxyquinoline bivalent metal complex BaQ_2_** is proposed. The visible‐light‐excited luminescent material has a yield of 99.6% and can photoluminesce under 408 nm visible light. The scanning electron microscopy analyses show that the mechanochemical activation method can be used to obtain BaQ_2_, which has better crystal forms, more uniform and smooth shapes, and less grain size.

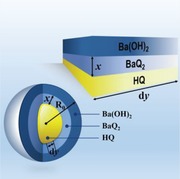

## 1 Introduction

8‐Hydroxyquinoline bivalent metal complex (MQ_2_) is an attractive kind of luminescent material,^1^ which has broad application prospects.^2^, ^3^ MQ_2_ (for example, 8‐hydroxyquinoline Barium, 8‐hydroxyquinoline magnesium,^4^, ^5^ 8‐hydroxyquinoline calcium,^6^, ^7^ and 8‐hydroxyquinoline zinc^8^, ^9^, ^10^, ^11^) has many advantages, such as simple synthesis conditions,^12^, ^13^ high luminous efficiency,^14^, ^15^, ^16^ and high thermal stability.^17^ Many scholars have done in‐depth research^18^, ^19^ focused on extending application potential^20^, ^21^ of MQ_2_. At present, liquid‐phase method^22^, ^23^ is the main method to synthesize MQ_2_. Most of liquid‐phase methods used organic solvents that pollute the environment and harm the human body, such as methanol,^24^ acetone,^25^ ether,^26^ and acetic acid.^27^ Another disadvantage of liquid‐phase method is low yield.^28^ The disadvantage is that MQ_2_ can only be excited under ultraviolet light by liquid‐phase method.^4^, ^5^, ^6^, ^7^, ^8^, ^9^, ^10^, ^11^ This spends a massive amount of energy and severely limits the scope of application of MQ_2_. Compared with ultraviolet light, visible light have the advantages of lower biotoxicity, easier operating, and easier obtaining. Therefore, to excite MQ_2_ by visible light and to ensure that it keeps high luminous efficiency is the focus and difficulty of current research on this product. However, the existing synthesis methods have not been able to synthesize MQ_2_, which can photoluminescent by visible light. This study presents a new synthesize method to overcome this problem—that is, the mechanochemical activation method.

The mechanochemical activation method is compared with the traditional liquid‐phase method. First, there is no need to use organic solvents in the mechanochemical activation method and it is environmentally friendly. Second, because there is no chemical equilibrium, the yield of the mechanochemical activation method is much higher than that of the traditional liquid‐phase method. Third, smaller size BaQ_2_ can be synthesized by mechanization activation method. The mechanochemical activation method is compared with the traditional solid‐phase method. First, the mechanochemical activation method makes the reactants contact in the molecular level and it increases the contact area between the reactants. Second the mechanochemical activation method reduces the heat energy of the solid‐phase reaction, so that it can react under the condition of low heat.

This study demonstrates that the mechanochemical activation method can synthesize the BaQ_2_ that has a yield of 99.6% and can photoluminescent under 408 nm visible light. Findings from this study provide a new perspective on synthesis of the visible‐light‐excited luminescent material MQ_2_ and enhance the prospects in the field of luminescent materials.

Visible‐light‐excited 8‐hydroxyquinoline bivalent metal complex can not only be used in the field of luminescent materials but also has many other potential application prospects.^29^ Visible‐light‐excited 8‐hydroxyquinoline bivalent metal complex could be useful in terms of solar energy and would likely become a new class of energy materials^30^ and functional materials.^31^, ^32^

## 2 Results

### 2.1 FTIR Spectra Analysis

**Figure 1:**
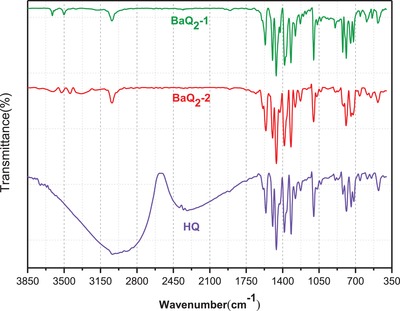
Infrared absorption spectra of 8‐hydroxyquinoline, BaQ_2_‐1 and BaQ_2_‐2.

The Fourier transform infrared (FTIR) spectra of BaQ_2_, which was synthesized by mechanochemical activation method (BaQ_2_‐1), the FTIR spectra of BaQ_2_, which was synthesized by liquid‐phase method (BaQ_2_‐2), and the FTIR spectra of the 8‐hydroxyquinoline (HQ) are shown in **Figure** **1**. Peaks in 3600–3300 cm^−1^ are ascribed to the fundamental stretching of O—H. The —OH of BaQ_2_‐1 may come from water in diluent KBr. Bands around 3039 cm^−1^ are due to C—C of 8‐hydroxyquinoline ring. The absorption peaks of C=C of 8‐hydroxyquinoline ring are observed at 1560, 1589, 1496, and 1460 cm^−1^. The peak in 1382 cm^−1^ is assigned to C—N. Because the hydroxyquinoline ring has aromaticity, it can trigger conjugated effect of π electron. And the conjugated effect makes the C—N stronger, so the peak moves to higher numerical value. The characteristic absorption peaks of BaQ_2_ are observed at 659, 549, and 484 cm^−1^. They are ascribed to Ba—O and Ba—N.

### 2.2 X‐Ray Diffraction Characterization

**Figure 2:**
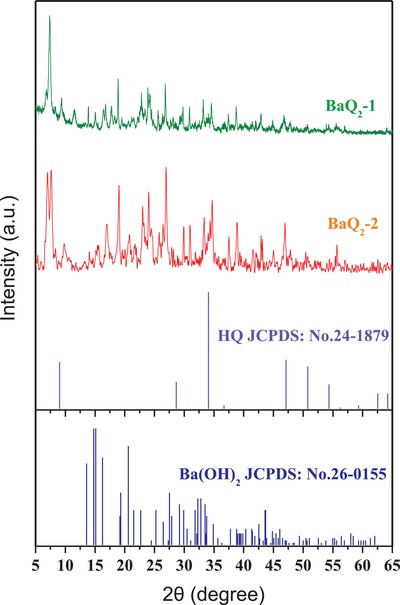
XRD patterns of 8‐hydroxyquinoline, Ba(OH)_2_, BaQ_2_‐1, and BaQ_2_‐2.

The X‐ray diffraction patterns of BaQ_2_‐1 and BaQ_2_‐2 are shown in **Figure** **2**. Comparing BaQ_2_‐1 with JCPDS standard card 24‐1879 of HQ and BaQ_2_‐1 with JCPDS standard card 26‐0155 of Ba(OH)_2_. They show that BaQ_2_ was synthesized successfully. Comparing BaQ_2_‐1 with BaQ_2_‐2, it shows that peaks of BaQ_2_‐1 are higher and more speculate than BaQ_2_‐2 and BaQ_2_‐1 has less impurity peaks, less full width at half maxima and less peak area then BaQ_2_‐2. It means that BaQ_2_‐1 has higher crystallinity, smaller particle size, and higher purity than BaQ_2_‐2. The results show that using mechanochemical activation method can get BaQ_2_, which has high crystallinity, small particle size, and high purity. According to Scherrer equation, the mean particle size is 56 nm. The lattice parameters of BaQ_2_‐1 cubic structure calculated using JADE 8.0 program are *a* = *b* = *c* = 5.34 Å. These results are consistent with synthesis mechanism of mechanochemical activation method below.

### 2.3 Photoelectron Spectroscopy Analysis

**Figure 3:**
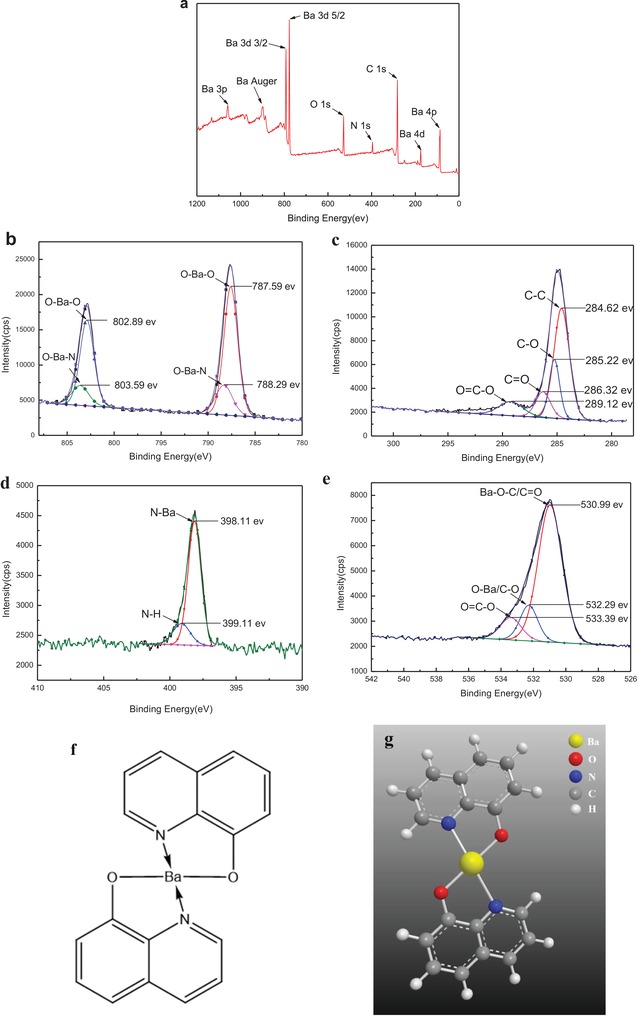
Photoelectron spectroscopy analysis of BaQ_2_‐1. a) XPS spectra of survey scan of BaQ_2_‐1. b) XPS spectra of Ba 3d of BaQ_2_‐1. c) XPS spectra of C 1s of BaQ_2_‐1. d) XPS spectra of N 1s of BaQ_2_‐1. e) XPS spectra of O 1s of BaQ_2_‐1. f) Molecular formula of BaQ_2_‐1. g) Structural model of BaQ_2_‐1.

The X‐ray photoelectron spectroscopy (XPS) of survey scan of BaQ_2_‐1 is shown in **Figure** **3**a. Consulting NIST X‐ray photoelectron spectroscopy database and referring the survey scan of BaQ_2_‐1, we can know that BaQ_2_‐1 has barium, nitrogen, carbon, and oxygen. The Auger line of Ba is on 898.0 eV and it does not shift. It shows that the valence of Ba of BaQ_2_‐1 has not changed. The survey scan of BaQ_2_‐1 conforms to BaQ_2_.

The XPS spectra detail scan of BaQ_2_‐1 is shown in Figure 3b–e. During fitting limit energy interval of spin coupling and spin splitting, number of pear splitting and ratio of pear areas, full width at half maxima and ratio of Gaussian‐ lineshape and Lorentzian‐lineshape. XPS spectra of Ba 3d of BaQ_2_‐1 decomposes Ba 3d into four peaks (Figure 3b). The binding energy (BE) of these four peaks are 803.59, 802.89, 788.29, and 787.59 eV, respectively. The peaks in 802.89 eV (3d 3/2) and 787.59 eV (3d 5/2) can assign to O—Ba—O. And the peaks in 803.59 eV (3d 3/2) and 788.29 eV (3d 5/2) can assign to O—Ba—N. XPS spectra of C 1s of BaQ_2_‐1 decomposes C 1s into four peaks (Figure 3c). The BE of these four peaks are 284.62, 285.22, 286.32, and 289.12 eV. And the peaks can respectively assign to C—C, C—O, C=O, and O=C—O. XPS spectra of N 1s of BaQ_2_‐1 decomposes N 1s into two peaks (Figure 3d). The BE of these two peaks are 398.11 and 399.11 eV. And the peaks can respectively assign to N—Ba and N—H. XPS spectra of O 1s of BaQ_2_‐1 decomposes O 1s into three peaks (Figure 3e). The BE of these three peaks are 530.99, 532.29, and 533.39 eV, respectively. And the peaks can respectively assign to Ba—O—C/C=O, O—Ba/C=O, and O=C—O. The results showed that BaQ_2_ was synthesized successfully. The molecular formula and structural model of BaQ_2_‐1 were shown in Figure 3f,g according to these results.

### 2.4 Scanning Electron Microscope (SEM) Characterization and Energy Dispersive Spectroscopy (EDS) Analyzation

**Figure 4:**
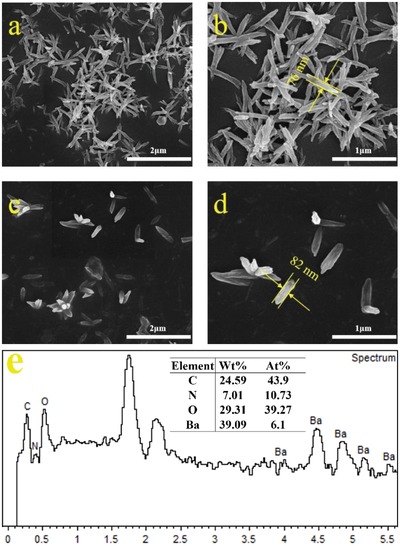
a–d) SEM image of BaQ_2_‐1. e) EDS image of BaQ_2_‐1.

**Figure 5:**
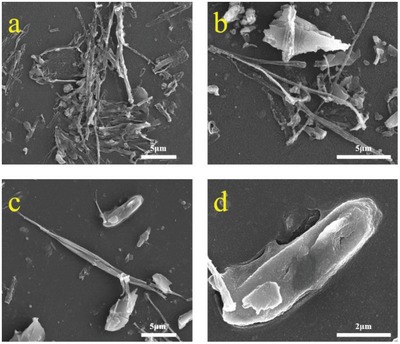
SEM image of BaQ_2_‐2.

The scanning electron microscope image of BaQ_2_‐1 is shown in **Figure** **4**. It shows that the morphology of BaQ_2_‐1 is regular and the surface of BaQ_2_‐1 is uniform and smooth. SEM shows that the morphology of single BaQ_2_‐1 crystal is short clavate, in which the average diameter is about 90 nm. Some BaQ_2_‐1 crystals get together as coralline. The scanning electron microscope image of BaQ_2_‐2 is shown in **Figure** **5**. SEM shows that the morphology of single BaQ_2_‐2 is in irregular shape and BaQ_2_‐2 assembles more loosely. The diameter of clavate crystal of BaQ_2_‐2 is from 400 to 900 nm. Comparing SEM images between BaQ_2_‐1 and BaQ_2_‐2. Results show that using the mechanochemical activation method can get BaQ_2_, which has better crystal forms, more uniform and smooth shapes and less grain size. The results are consistent with synthesis mechanism of theoretical studies of mechanochemical activation method below. It can be seen from the EDS image (Figure 4e) of BaQ_2_‐1 that the C, N, O, and Ba elements exist in it, and the weight percentage and atomic percentage of each element are basically consistent with the chemical formula of BaQ_2_.

### 2.5 Fluorescence Analysis

**Figure 6:**
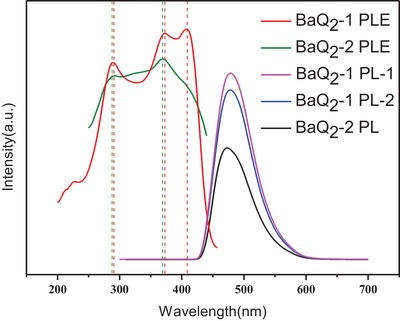
Fluorescence spectra of BaQ_2_‐1 and BaQ_2_‐2 samples. The BaQ_2_‐1 PLE is the excitation spectra of BaQ_2_‐1; The BaQ_2_‐2 PLE is the excitation spectra of BaQ_2_‐2; The BaQ_2_‐1 PL‐1 is the emission spectra of BaQ_2_‐1 at 408 nm exciting light; The BaQ_2_‐1 PL‐2 is the emission spectra of BaQ_2_‐1 at 372 nm exciting light; The BaQ_2_‐2 PL is the emission spectra of BaQ_2_‐2 at 369 nm exciting light.

The excitation spectra and emission spectra of BaQ_2_‐1 and BaQ_2_‐2 are shown in **Figure** **6**. The excitation spectra of them is obtained by scanning the full wavelength. The excitation spectrum of BaQ_2_‐1 appears in three excitation peaks at 289, 372, and 408 nm, respectively. The maximum excitation wavelength of BaQ_2_‐1 is 408 nm. The excitation spectrum of BaQ_2_‐2 appears in two excitation peaks at 287 and 369 nm, respectively. The maximum excitation wavelength of BaQ_2_‐2 is 369 nm. Comparing the excitation spectrum of BaQ_2_‐1 and the excitation spectrum of BaQ_2_‐2. It shows that from 280 to 370 nm excitation peaks of BaQ_2_‐1 are higher than excitation peaks of BaQ_2_‐2 and have better peak shape and less full width at half maxima. It shows that BaQ_2_‐1 has better selectivity than BaQ_2_‐2. The excitation spectrum of BaQ_2_‐2 begins to decline after 370 nm and decline very fast after 400 nm. The excitation spectrum of BaQ_2_‐1 has a strong peak at 408 nm. It shows that BaQ_2_‐1 can be excited by visible light.

The emission spectrums show that the emission spectrum of BaQ_2_‐1 at 408 nm has higher luminous intensity than 370 nm. And the maximum emission wavelength of BaQ_2_‐1 is 475 nm, which belongs to blue laser. Comparing the emission spectrum of BaQ_2_‐1 and BaQ_2_‐2. The luminous intensity at 370 nm of BaQ_2_‐1 is 1.5 times that of BaQ_2_‐2. The luminous intensity at maximum emission wavelength of BaQ_2_‐1 is 1.7 times that of BaQ_2_‐2. It shows that BaQ_2_‐1 has higher luminous efficiency than BaQ_2_‐2. The results show that using the mechanochemical activation method can get visible‐light‐excited BaQ_2_ at 408 nm visible light and has higher luminous efficiency. These results are consistent with the theoretical studies of the luminescence mechanism of BaQ_2_ below.

## 3 Theoretical Studies

### 3.1 Synthesis Mechanism of Mechanochemical Activation Method

The essence of mechanochemical activation method is using mechanical force to activate reactants before the solid‐phase reaction. Mechanochemical activation method can decrease the radiuses of solid particles of reactants, increase the contact areas between reactants, and make reactants contact uniformly on a molecular‐level by mechanical force. Mechanochemical activation method can also activate reactants to reduce the thermal energy of the solid‐phase reaction needed. So that reactants can react at low‐heating temperature and the reaction time can be short.

### 3.2 Thermodynamic Principles of Mechanochemical Activation Method

The equation of definition of the Gibbs free energy change (Δ*G*) of thermodynamic functions in a chemical reaction can be written as Equation (1)

(1) ΔG = ΔH − TΔS

According to Equation (1), Δ*S* of the solid‐phase reaction is small enough to ignore. So, Δ*G* of the pure solid‐phase reaction is only relevant for Δ*H*. If ∆*H* is less than zero, then Δ*G* would be less than zero, too. That means that once the pure solid‐phase reaction takes place, Δ*G* would always be less than zero. It causes that there is no chemical equilibrium in the pure solid‐phase reaction and it goes on to the end as soon as the reaction takes place. The rate of production of pure solid‐phase reaction is 100%. After mechanical activation, the chemical reactivity of 8‐hydroxyquinoline and barium hydroxide (Ba(OH)_2_) is enhanced. They cause that the reaction of 8‐hydroxyquinoline and barium hydroxide can take place in the low heat and the rate of production of mechanochemical activation method is high.

### 3.3 Dynamic Principles of Mechanochemical Activation Method

**Figure 7:**
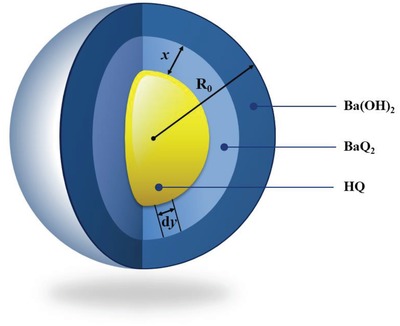
The microcosmic dynamic model of the reaction of HQ and Ba(OH)_2_.

The solid‐phase reaction consists of several simple physical and chemical processes. This research divided the reaction of HQ and Ba(OH)_2_ into two processes. One is chemical reaction process of HQ and Ba(OH)_2_ on contact surface. The other is the diffusion process of Ba(OH)_2_ through the product layer. The microcosmic dynamic model of the reaction of HQ and Ba(OH)_2_ is built and shown in **Figure** **7**.

Ba(OH)_2_ is the diffusive phase, which is wrapped on the surface of HQ (Figure 7). HQ reacts with Ba(OH)_2_ and forms BaQ_2_ on the interface. For the further reaction, Ba(OH)_2_ needs to penetrate the product layer of BaQ_2_. So, the reaction takes place from the surface of HQ to the center. *R*_0_ is the radius of HQ at the beginning, and *x* is the thickness of the product layer. According to Equation (2), it shows the reaction rate on the surface

**Figure 8:**
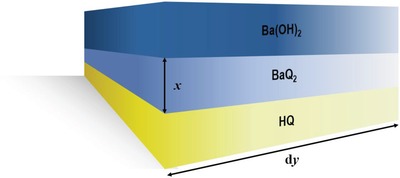
The dynamic model of Ba(OH)_2_ diffusing on d*y*.

(2) x = Kt

where *K* is the reaction rate constant. That is that *x* is only concerned with the kind of reactant and reaction time. Pick a short arc (d*y*) on the contact surface and enlarge it. The arc can regard as a short straight line because the arc is enough short. The dynamic model of Ba(OH)_2_ diffusing on d*y* is built (**Figure** **8**).

HQ is the diffusion medium and *x* is the diffusion path. According to the Fick's first law, the equation on steady state diffusion can be written as

(3) $$J_{i}=-D\cdot \frac{\Delta c_{i}}{\Delta x}$$

where *J*_i_ is the diffusion velocity of Ba(OH)_2_; *D* is diffusion coefficient; ∆*c*_i_ is the concentration difference of Ba(OH)_2_; ∆*x* is the diffusion path of Ba(OH)_2_. According to Equation (3), the main factors affecting the rate of diffusion on steady state diffusion are physical property and material concentration. The smaller the particle radius is, the more regular particle shape will be. The larger the dispersion of particles is, the smaller the diffusional resistance will be. And finally, the greater the diffusion coefficient is, the faster the diffusion rate will be. This study grinds HQ and Ba(OH)_2_ by mechanical force. It can effectively reduce particle radius and mix particles evenly. It causes that the diffusion rate is increased and the diffusion time is decreased. The diffusion coefficient of solid in a solid is much smaller than other media. That is, the rate of diffusion is relatively slower. According to the theory of rate‐determining step, the rate of reaction between HQ and Ba(OH)_2_ is determined by the rate of diffusion. That is the mechanochemical activation method increases the rate of diffusion and then increased the reaction rate of the whole reaction.

The conclusion of appeal can also be confirmed by the theory of solid‐state reaction rate (α). α means that the ratio of the reactive volume to the original volume. α can be written as Equation (4)

(4) $$\alpha \;=\;\frac{R_{0}^{3}\;-\;{\left(R_{0}\;-\;x\right)}^{3}}{R_{0}^{3}}$$

And Equation (4) can transform as Equation (5)

(5) $$\alpha \;=\;1\;-\;\frac{{\left(R_{0}-\;Kt\right)}^{3}}{R_{0}^{3}}$$

Find the limit at the same time on both sides of Equation (5) as Equation (6)

(6) $$\underset{R_{0}\to 0}{\lim}\alpha \;=\underset{R_{0}\to 0}{\lim}\;\left[1-\frac{{\left(R_{0}-Kt\right)}^{3}}{R_{0}^{3}}\right]=\;1$$

According to Equation (6) the smaller *R*_0_ is, the larger α will be. That is the smaller the radius of 8‐hydroxyquinoline particles is, the faster the reaction rate will be.

Based on appeal analysis, mechanochemical activation method can effectively increase the rate of reaction between HQ and Ba(OH)_2_.

### 3.4 Luminescence Mechanism of BaQ_2_

BaQ_2_ belongs to metal ion perturbation ligand luminescence. 8‐hydroxyquinoline ring absorbs energy and induces π–π^+^ transition. And then the de‐excitation of the exciton go by luminescing and vibrational relaxation. The addition of barium ions can increase molecular rigidity, reduce vibrational relaxation and increase luminous efficiency.

### 3.5 Excitation Spectrum Redshift of BaQ_2_

Lattice parameter expansion of BaQ_2_. The crystal is composed of crystallite and grain boundary, and the interplanar spacing comes from the contribution of crystallite and grain boundary. For general large‐sized crystals, the specific surface area increases and the surface atoms increase with the decrease of grain size. The coordination number of surface atoms is lower than internal atoms. It will cause the increase of dangling bond, increase of surface energy, decrease of atomic radius, and lattice parameter contraction. But there is no independent surface in the near nanometer crystal and the grain boundary energy is lower than the surface energy so the degree of lattice parameter contraction is much smaller. On the other hand, as the grain size decreases, because of the surface effect, the atomic spacing of the surface atoms is larger than the internal atoms. According to Equations (7) and (8) it can be concluded that the intermolecular force of surface atoms is smaller and the bond length is longer. The results of equations show that the lattice constant increases and the lattice parameter expansion

(7) $$U\;=\frac{B}{r^{12}}\;-\frac{A}{r^{6}}$$

(8) $$U_{e}=\;-\;\frac{kq\left(+\right)emq\left(-\right)e}{r_{0}}\;-\;\frac{kq\left(+\right)em\mu_{i}}{r_{0}^{2}}\;+\;\frac{kmB}{r_{0}^{9}}$$

where *U* is van der Waals force; *U*_e_ is coordinate covalent bonds energy. Third, the ordering of grain boundary is high, it will also lead to lattice parameter expansion.

### 3.6 Shrink of Bandgap and Decline of Zero‐Phonon Transition Energy of BaQ_2_

Some basic conclusions about electronic states in crystal materials can be deduced directly or inferred from the Kronig–Penney model. Based on the Bloch theorem, the analytic solution of the Schrodinger equation is obtained by using Kronig–Penney model, and the transcendental equation which can determine the electron energy (*E*_e_) is obtained. The simplified result shows on Equations (9) and (10).

(9) $$P\frac{\sin\alpha a}{\alpha a}\;+\;\cos\alpha a\;=\;\cos k\;a$$

(10) $$\frac{2mE_{e}}{\hbar^{2}}\;=\;\alpha^{2}$$

where *P* is a constant; *a* is periodic potential field; *k* is wave vector; α is related to *E*_e_. The value of *αa* is not arbitrary according to the Equation (9), and the range is limited by the in Equation (11)

(11) $$\left|P\frac{\sin\alpha a}{\alpha a}\;+\;\cos\alpha a\right|\leq 1$$

So simultaneous Equations (9) and (10) can be deduced that the relative volume deformation of periodic potential field is related to the specified energy levels (*E*)

(12) $$E\;=\;\frac{\hbar \alpha^{2}}{2m}\;=\;\frac{\hbar^{2}{\left(\alpha a\right)}^{2}}{2ma^{2}}$$

(13) $$E_{gn}=\frac{\hbar^{2}}{2ma^{2}}\;\left[{\left(\alpha a\right)}_{n+1}^{2}-\;(\alpha a)\prime_{n}^{2}\right]$$

Or

(14) $$E_{gn}\;=\;\frac{\hbar^{2}}{2m}\;\left[{\left(\alpha a\right)}_{n+1}^{2}\;-\;(\alpha a)\prime_{n}^{2}\right]v^{-\frac{2}{3}}$$

where (*αa*)_*n*+1_ and (αa)′ are constant value; *v* is unit cell volume; *E*_g*n*_ is the *n*th bandgap. Get Equations (13) and (14) by the integral of Equations (15) and (16)

(15) $$dE_{g}=\;-\frac{2}{3}E_{g}\frac{dv}{v}$$

(16) $$\Delta E_{g}=\;-\frac{2}{3}E_{g_{0}}\frac{\Delta v}{v_{0}}$$

where *E*_g_ is bandgap. The Equations (15) and (16) show the relation between *E*_g_ and relative volume deformation. That is the lattice parameter contraction will cause the increase of *E*_g_ and the lattice parameter expansion will cause the decrease of *E*_g_.

The Equations (15) and (16) can be further extended to the relation between specified energy levels (include the top of valence band and the bottom of conduction band) and relative volume deformation

(17) $$dE\;=\;-\frac{2}{3}E\frac{dv}{v}$$

Or

(18) $$\Delta E\;=\;-\frac{2}{3}E_{0}\frac{\Delta v}{v_{0}}$$

It means that the lattice parameter contraction will cause the increase of *E* and the lattice parameter expansion will cause the decrease of *E*.

The energy of transition of luminescence center from the ground state to the excited state is called charge‐transfer energy (*E*_CT_). According to Equation (19), *E*_CT_ consists of zero‐phonon energy (*E*_zp_) and vibrational energy (*E*_vib_)

(19) $$E_{\mathrm{CT}}\;=\;E_{\mathrm{zp}}\;+\;E_{\mathrm{vib}}$$

where *E*_zp_ is the energy of transition of one electron from the top of valence band to the bottom of conduction band. So as the decrease of *E*_g_, the energy between valence band and conduction band would decrease and result in the decrease of *E*_zp_. Similarly, *E*_CT_ would decrease. Finally, with the decrease of *E*_CT_, less energy needed for excite and result in red‐shifting of the excitation spectra.

This research uses mechanochemical activation method to synthesize BaQ_2_, which grain size is less than 100 nm. Base on the above inference the excitation spectra of BaQ_2_ redshift and the results are consistent with fluorescence spectrum of BaQ_2_.

### 3.7 New Excitation Peak of BaQ_2_ on Visible Region

**Figure 9:**
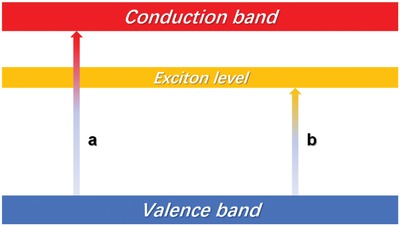
Where a is excite between valence band and conduction band; b is excite between valence band and exciton level.

On the one hand with the decrease of grain size, 8‐hydroxyquinoline barium's specific surface area, interface area, and the number of triple junction increase. And it leads to number of crystal defects, vacancy and vacancy cluster increase. The new crystal defects, vacancy or vacancy cluster increase would cause the new light absorption on visible region. On the other hand, because of the quantum confinement effect, the structure of boundary surface is unordered and the mean free path of an electron is short. It causes that the constraint of electron by the vacancy is weak and the probability of exciton formation is high. It results in high concentration of exciton. Finally, the high concentration of exciton would form a new and lower exciton level (**Figure** **9**). It can decline the energy of electron transition and form a new excitation peak on visible region.

### 3.8 The De‐Excitation Process of BaQ_2_ Exciton

**Figure 10:**
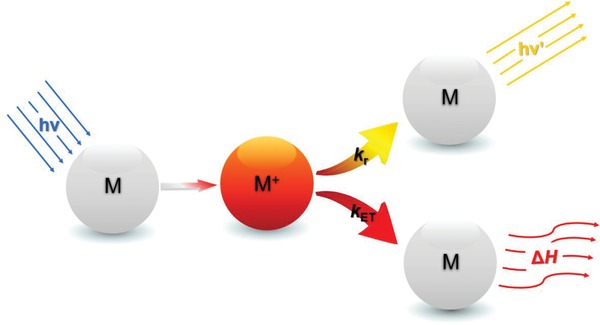
Where M is the ground state atom; M^+^ is the excited state atom; *k*_r_ is the rate constant of radiative decay; *k*_ET_ is the rate constant of radiationless decay.

The de‐excitation process of BaQ_2_ exciton consists of radiative decay process and radiationless decay process (**Figure** **10**). And radiationless decay process consists of Förster resonance energy transfer (FRET) and Dexter excitation transfer.

According to Equations (20) and (21), FRET and Dexter excitation transfer are all functions of distance between donor and acceptor

(20) $$k_{F\left(D\to A\right)}=\frac{K^{2}J\cdot 8.8\;\times \;10^{-28}}{n^{4}\omega r^{6}}$$

where *k*_F(D→A)_ is the rate constant of FRET; *K* is orientation factor; *n* is refractive indices of media; ω is radiative lifetime of energy donor; *r* is the distance between donor and acceptor; *J* is overlap integral of spectra

(21) $$k_{D\left(D\to A\right)}\propto \left(\frac{h}{2\pi }\right)P^{2}Je^{-\frac{2r}{L}}$$

where *k*_D(D→A)_ is the rate constant of Dexter excitation; *r* is the distance between donor and acceptor; *P* and *L* are constant. With the decrease of grain size of 8‐hydroxyquinoline barium crystal, the lattice parameter is expanded and the bond length is elongated. It causes the distance between donor and acceptor longer and *k*_ET_ would decline rapidly. Finally, radiationless decay process would reduce and result in the increase of fluorescence efficiency and the enhancing of luminous intensity. The results are consistent with the fluorescence spectrum of 8‐hydroxyquinoline barium.

## 4 Conclusion

This study proposes a new method for the synthesis 8‐hydroxyquinoline bivalent metal complex. We successfully synthesized BaQ_2_ that can be excited by visible light with a maximum excitation wavelength of 408 nm. We have explained the efficiency of the mechanochemical activation method proposed by this study, by thermodynamic principles, and dynamic principles. Our study is also explained by theoretical deduction that the maximum excitation wavelength of BaQ_2_ can be red shifted by decreasing the grain size. We expanded photoluminescence theory as it applies to visible‐light‐excited BaQ_2_ and further improved photoluminescence theory of 8‐hydroxyquinoline bivalent metal complex. These findings can provide a new perspective on the potential for using luminescent materials for energy conservation. The study of the special luminescent properties of nanomaterials is still in its infancy. Improving the accuracy of detection equipment and the diversity of detection methods can help to improve and verify theories.

## 5 Experimental Section

*Synthesis of BaQ_2_‐1 and BaQ_2_‐2*: 1.4053 g (0.0097 mol) of 8‐hydroxyquinoline (AR), 1.4869 g (0.0047 mol) of barium hydroxide (AR) were placed in the Planetary ball mill of Pulverisette 7 of FRITSCH company in Germany and grind for 1 h. The product powder was put into a 100 mL beaker. The beaker was put into a vacuum drying oven. The temperature of the vacuum drying oven was raised to 90 °C and kept at 1 h. Then the temperature was raised to 100 °C and kept at 0.5 h. Then it was cooled to room temperature. Finally, 1.9982 g BaQ_2_‐1 was obtained at yield 99.60%. BaQ_2_‐2 was obtained by liquid‐phase method.^22^

*Characterization of BaQ_2_‐1 and BaQ_2_‐2*: The infrared radiation spectra analysis (IR) of BaQ_2_‐1 and BaQ_2_‐2 was recorded using KBr pellets in the range of 4000–400 cm^−1^ on a Mattson Alpha‐Centauri spectrometer. XRD of BaQ_2_‐1 and BaQ_2_‐2 was recorded on a Rigaku D‐MAX 2550 radiation (λ = 0.15417 nm) with 2θ ranging from 5° to 70°. XPS with Al Kα radiation (*hv* = 1486.6 eV) as the photo source was used to investigate the surface properties of BaQ_2_‐1. SEM and EDS were done on a JEOL JSM‐6700F SEM microscope Japan. The photoluminescence properties of BaQ_2_‐1 and BaQ_2_‐2 were recorded using Hitachi F‐7000 fluorescence spectrometer with 150 W monochromatic xenon lamp as excitation source.

## Conflict of Interest

The authors declare no conflict of interest.

## Supporting information

Supplementary
