# Supplementary material for: Green Synthesis of 8‐Hydroxyquinoline Barium as Visible‐Light‐Excited Luminescent Material Using Mechanochemical Activation Method
Source: Glob Chall. 2019 Sep 6;3(12):1900052. doi: 10.1002/gch2.201900052 (PMC6888826; doi:10.1002/gch2.201900052)
Supplement: Supplementary file 1 — Supplementary [file GCH2-3-na-s001.pdf]

Copyright WILEY-VCH Verlag GmbH & Co. KGaA, 69469 Weinheim, Germany, 2019.

Supporting Information

for *Global Challenges,* DOI: 10.1002/gch2.201900052

Green Synthesis of 8-Hydroxyquinoline Barium as Visible-Light-Excited Luminescent Material Using Mechanochemical Activation Method

Junchen Liu, Xueming Zhong, Yuna Xu, and Yanrong Li*

Supporting Information

Green Synthesis of 8-hydroxyquinoline Barium as Visible-light-excited Luminescent Material Using Mechanochemical Activation Method

Junchen Liu, Xueming Zhong, Yuna Xu, Yanrong Li*

Fluorescence Analysis

The photoluminescence properties of BaQ_2-1 and BaQ_2-2 were recorded using Hitachi F-7000 fluorescence spectrometer with 150 W monochromatic xenon lamp as excitation source. The details of the fluorescence spectrometer we used is shown (**Table S1**). Select 477 nm for EM WL, 200 nm for EX Start WL and 500 nm for EX End WL to determinate the excitation spectra of BaQ_2-1 (BaQ_2-1 PLE) and BaQ_2-2 (BaQ_2-2 PLE). Select 408 nm for EX WL, 300 nm for EM Start WL and 700 nm for EM End WL to determinate the emission spectra of BaQ_2-1 in 408 nm exciting light (BaQ_2-1 PL-1).Select 372 nm for EX WL, 310 nm for EM Start WL and 700 nm for EM End WL to determinate the emission spectra of BaQ_2-1 in 372 nm exciting light (BaQ_2-1 PL-2). Select 369 nm for EX WL, 200 nm for EM Start WL and 700 nm for EM End WL to determinate the emission spectra of BaQ_2-2 in 369 nm exciting light (BaQ_2-2 PL).

Table S1. The details of the fluorescence spectrometer.

SPECTROMETER	Hitachi Model F-7000 FL Spectrophotometer
Serial Number	2130-007
ROM Version	5J14000 06
Scan speed	1200 nm/min
Delay	0.3 s
EX Slit	5.0 nm
EM Slit	5.0 nm
PMT Voltage	400 V
Response	0.5 s
Corrected spectra	Off
